# Supplementary material for: Longitudinal assessment of DNA repair signature trajectory in prodromal versus established Parkinson’s disease
Source: NPJ Parkinsons Dis. 2025 Dec 5;11:349. doi: 10.1038/s41531-025-01194-7 (PMC12680741; doi:10.1038/s41531-025-01194-7)
Supplement: Supplementary file 1 — Supplementary Information [file 41531_2025_1194_MOESM1_ESM.pdf]

Title: Supplementary Figure S1: Expression Trajectories of Key Genes

Caption: Gene expression trajectories highlighting the most important genes, including repair-related and ISR-associated genes.

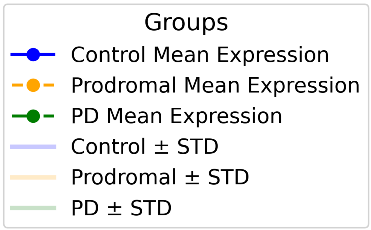

Repair genes

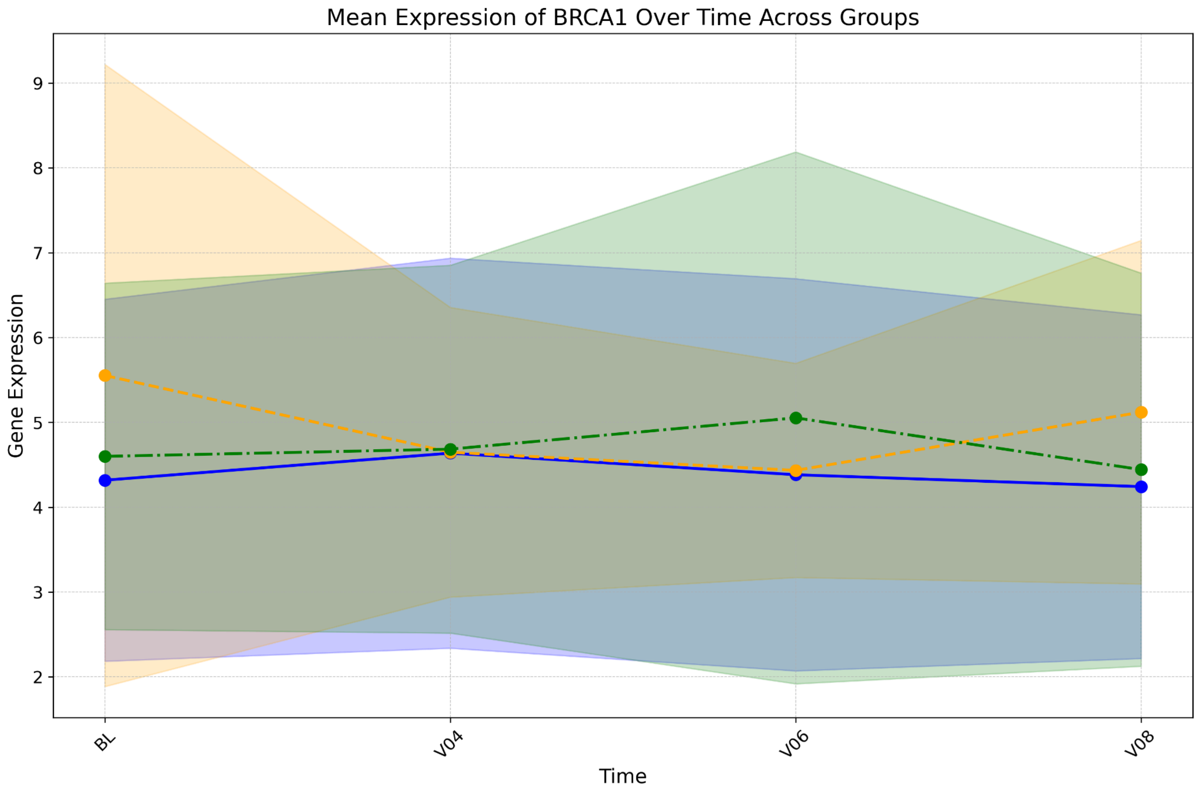

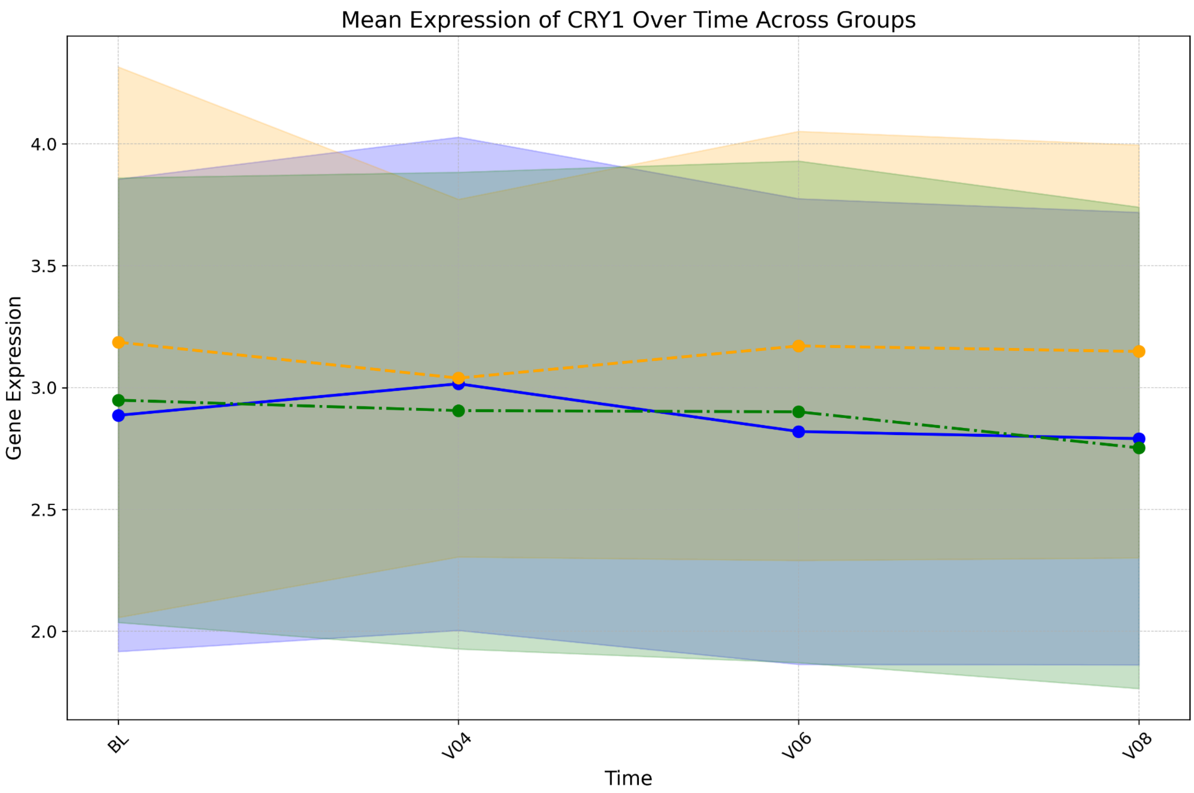
Mean Expression of CRY1 Over Time Across Groups
Gene Expression
4.0
3.5
3.0
2.5
2.0
BL
V04
V06
V08
Time

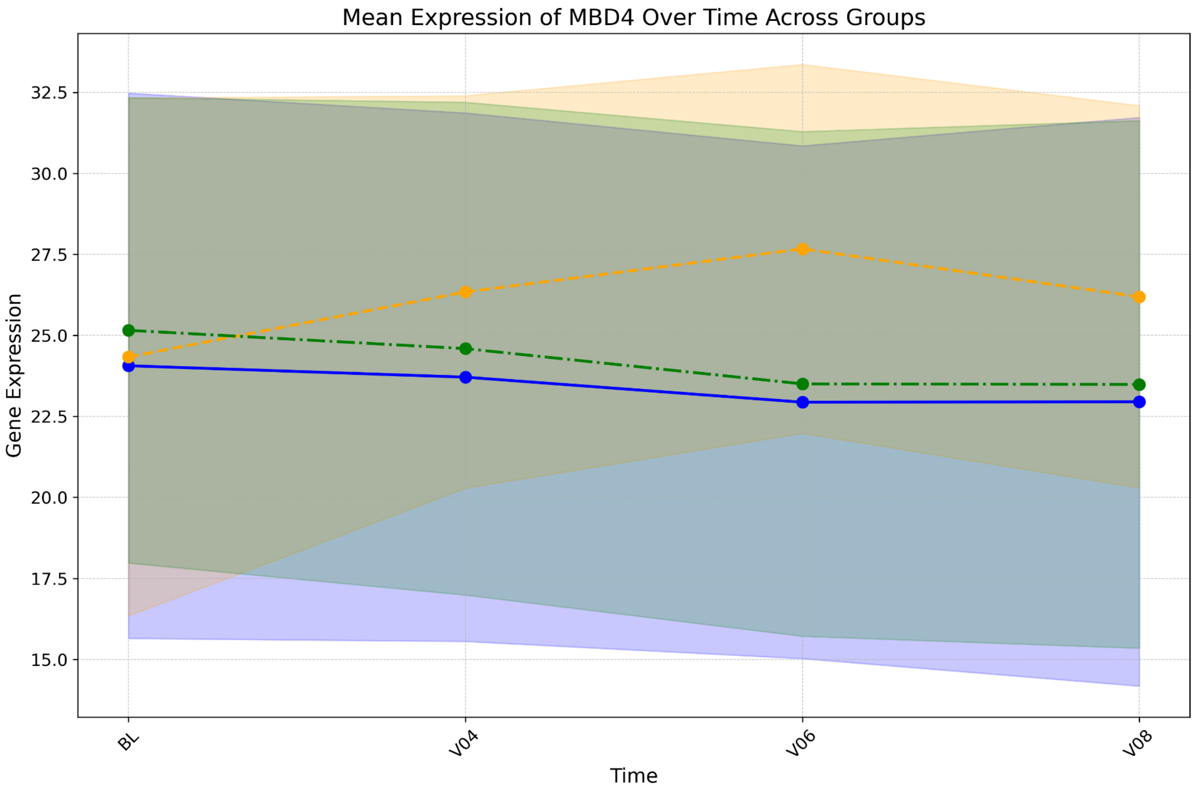
Mean Expression of MBD4 Over Time Across Groups
Gene Expression
32.5
30.0
27.5
25.0
22.5
20.0
17.5
15.0
BL
V04
V06
V08
Time

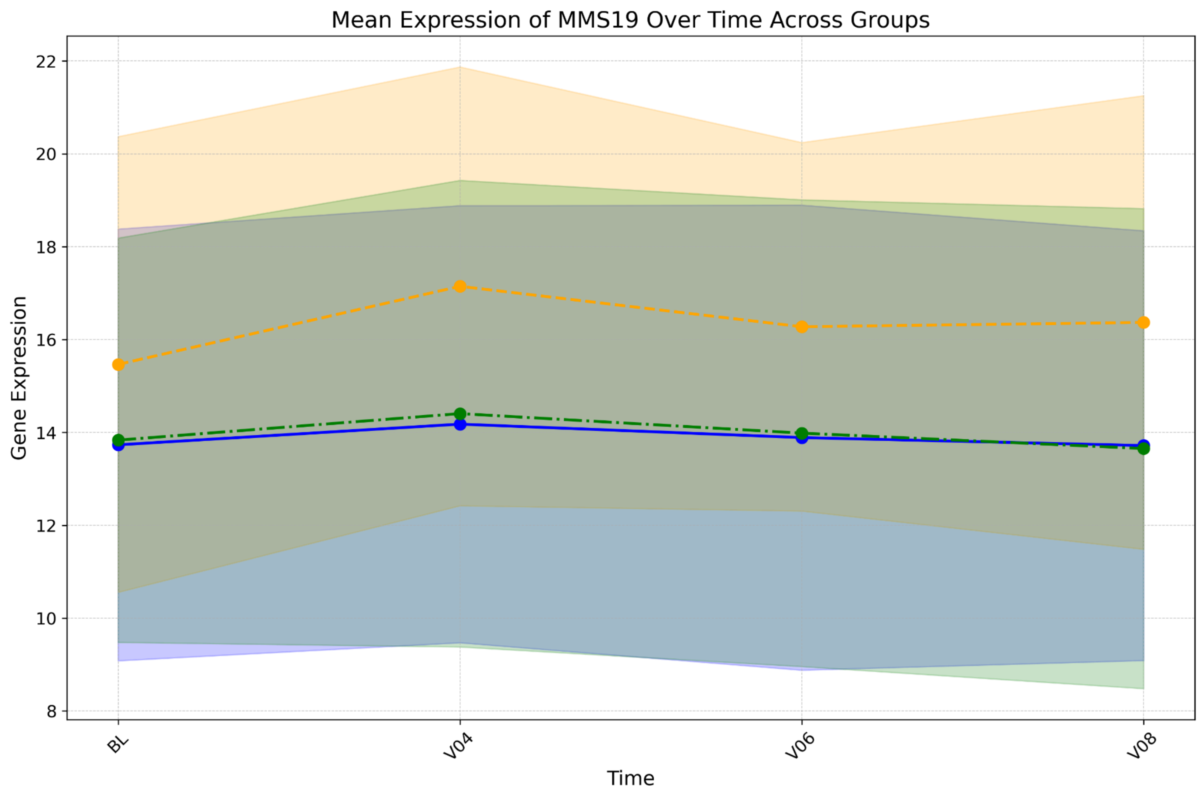

Mean Expression of MMS19 Over Time Across Groups
Gene Expression
22
20
18
16
14
12
10
8
BL
V04
V06
V08
Time

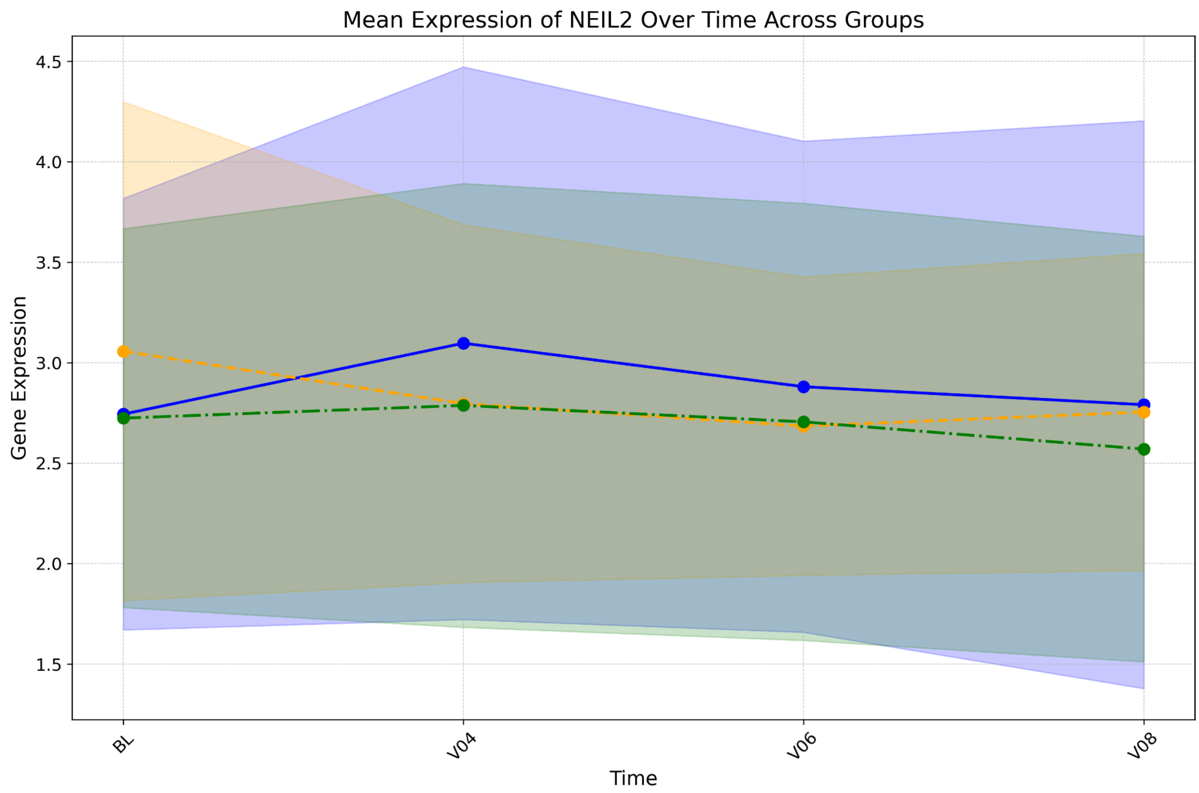

Mean Expression of NEIL2 Over Time Across Groups
Gene Expression
4.5
4.0
3.5
3.0
2.5
2.0
1.5
BL
V04
V06
V08
Time

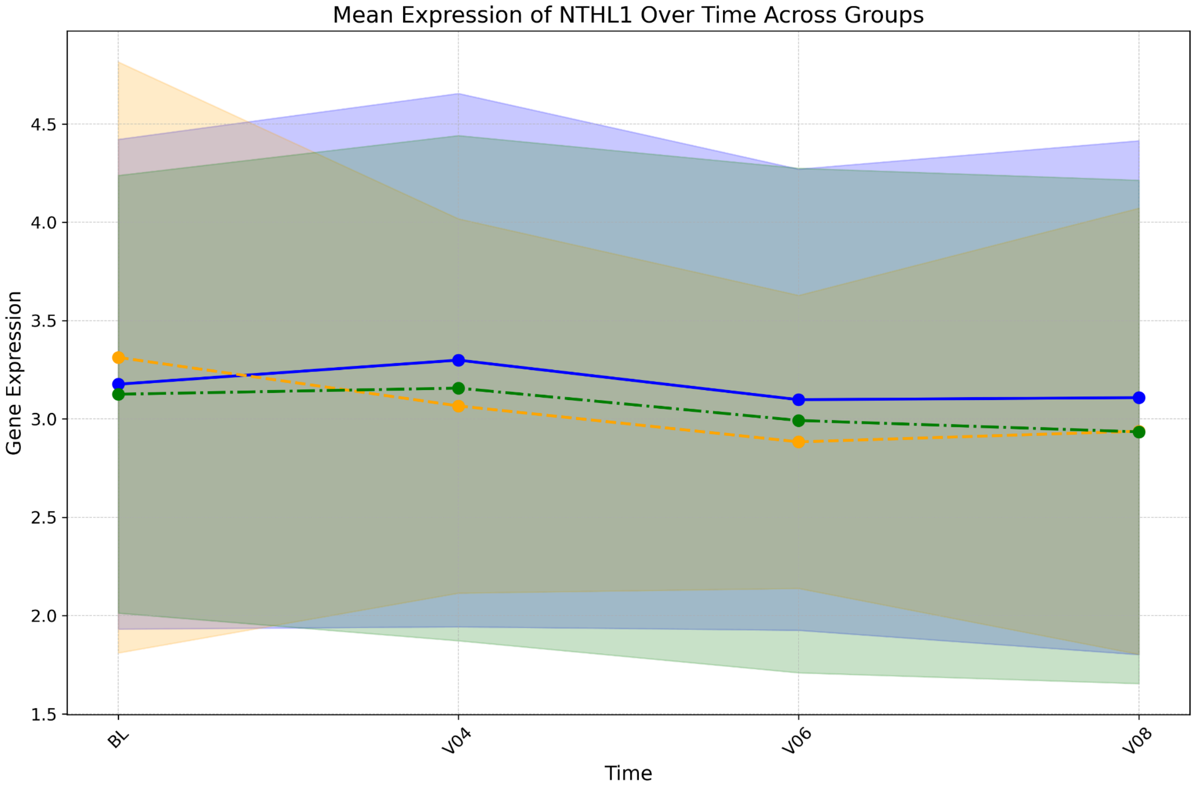

Mean Expression of NTHL1 Over Time Across Groups
Gene Expression
4.5
4.0
3.5
3.0
2.5
2.0
1.5
BL
V04
V06
V08
Time

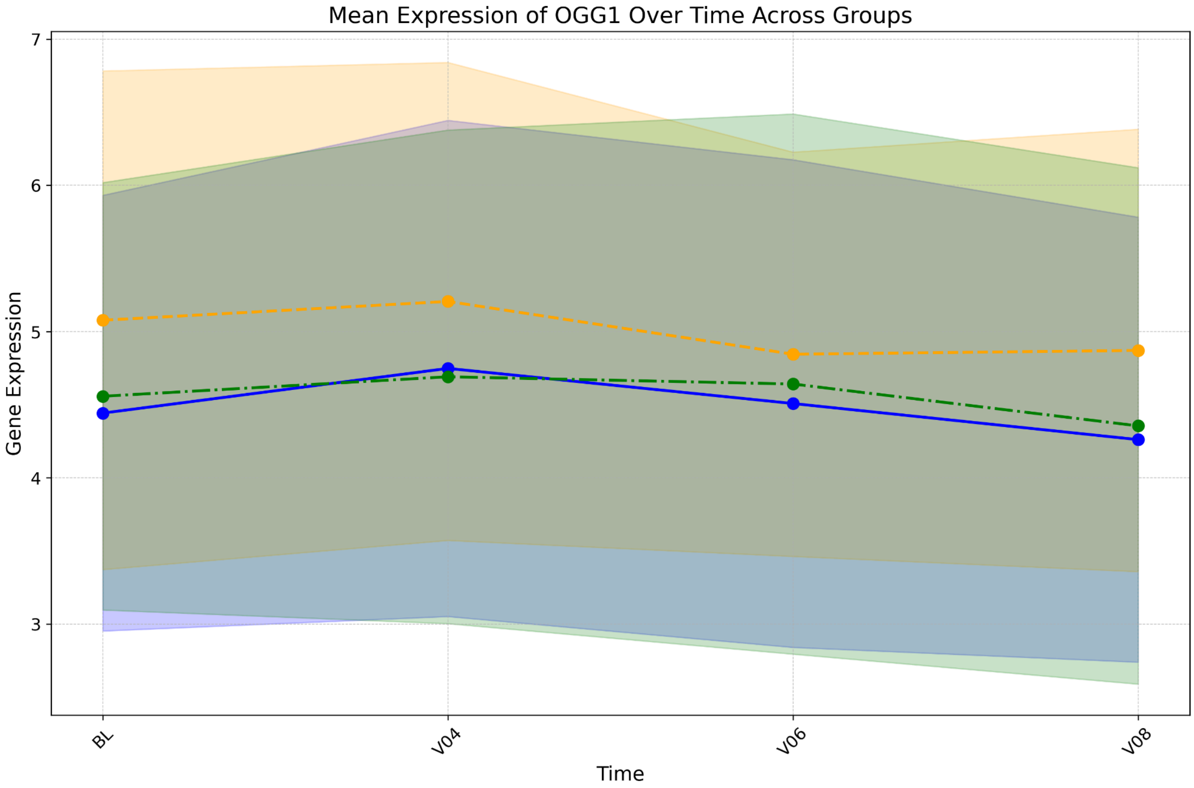

Mean Expression of OGG1 Over Time Across Groups
Gene Expression
7
6
5
4
3
BL
V04
V06
V08
Time

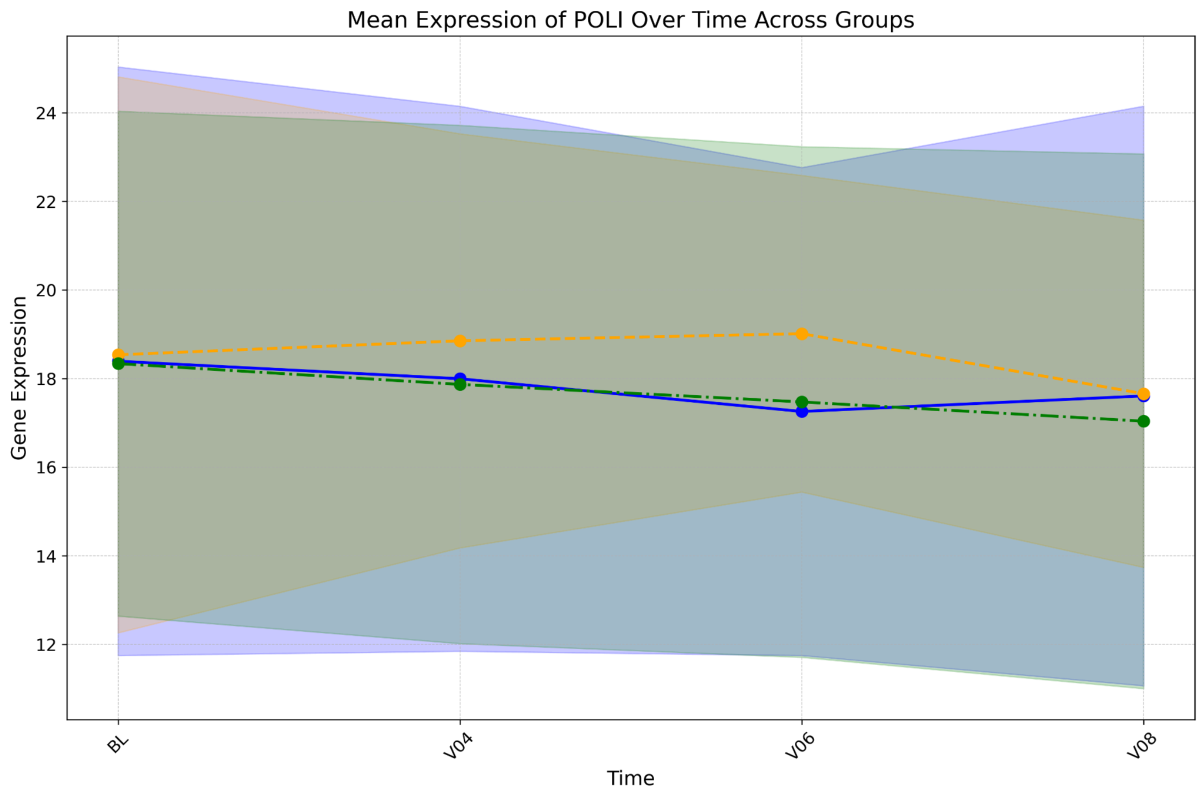
Mean Expression of POLI Over Time Across Groups
Gene Expression
24
22
20
18
16
14
12
BL
V04
V06
V08
Time

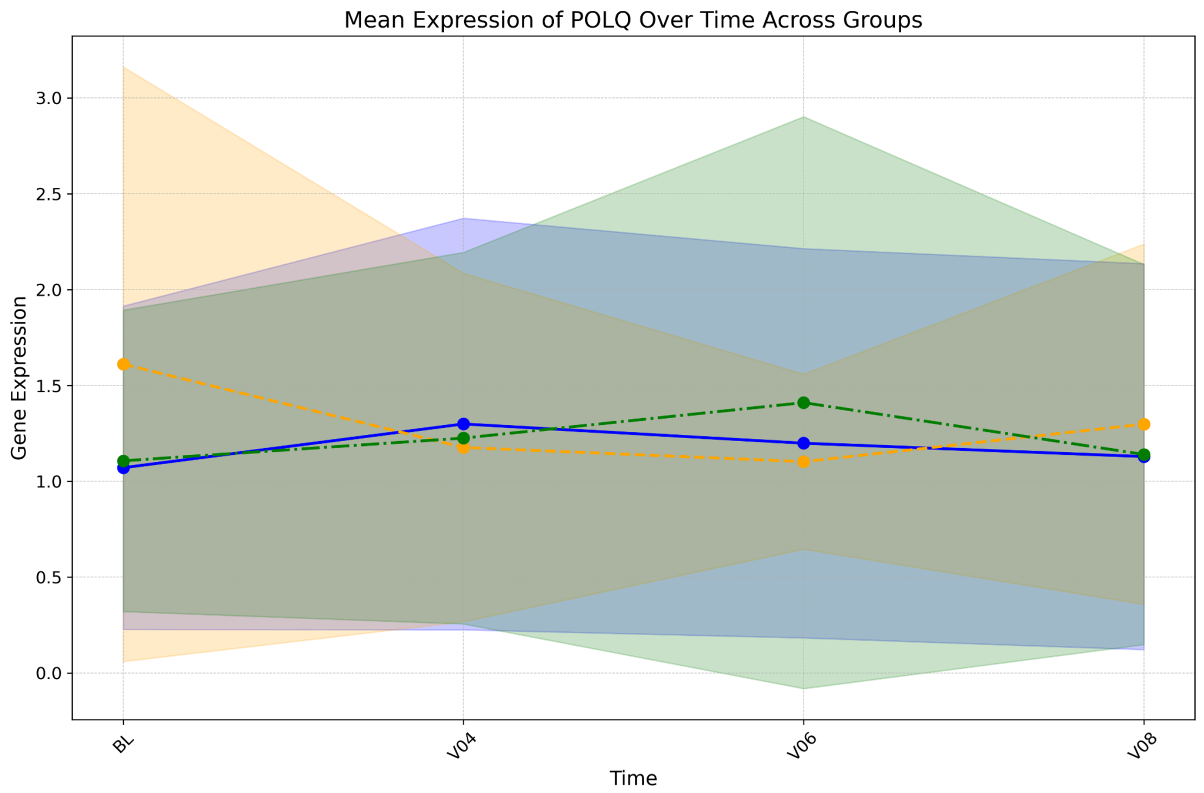
Mean Expression of POLQ Over Time Across Groups
Gene Expression
3.0
2.5
2.0
1.5
1.0
0.5
0.0
BL
V04
V06
V08
Time

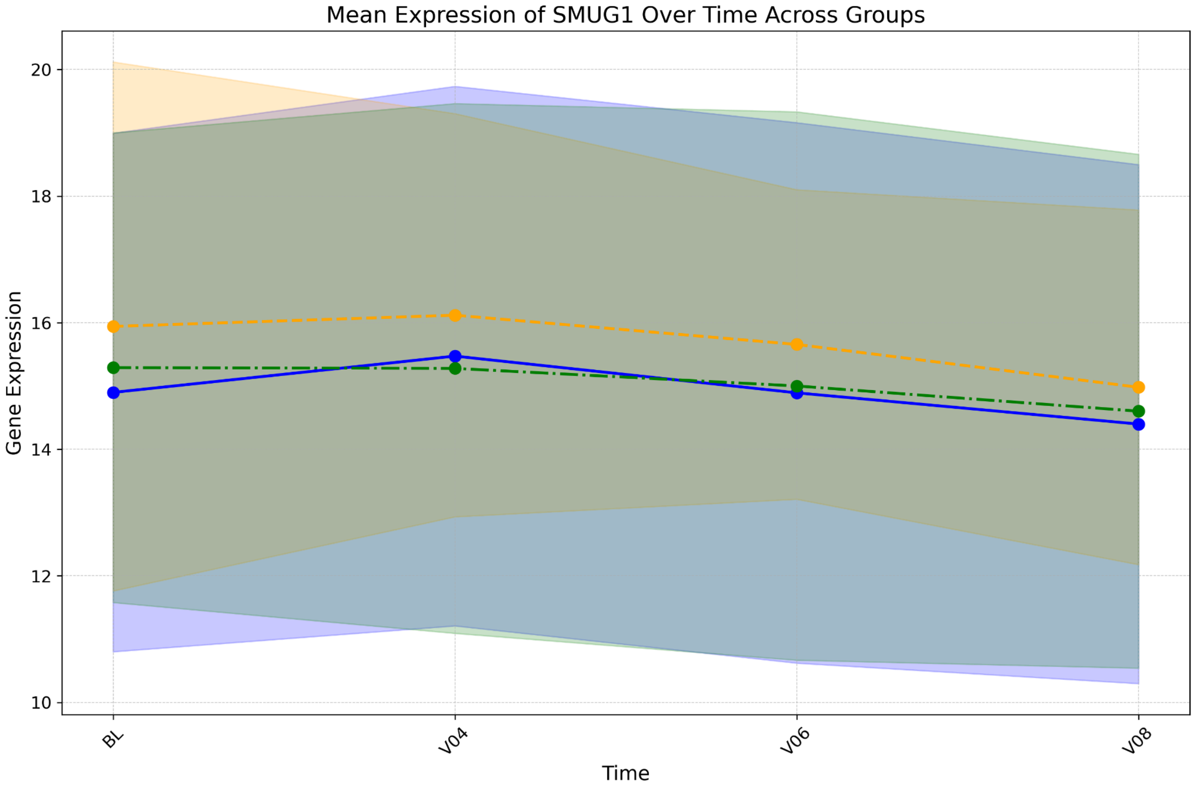

Mean Expression of SMUG1 Over Time Across Groups
Gene Expression
Time
BL
V04
V06
V08
10
12
14
16
18
20

ISR

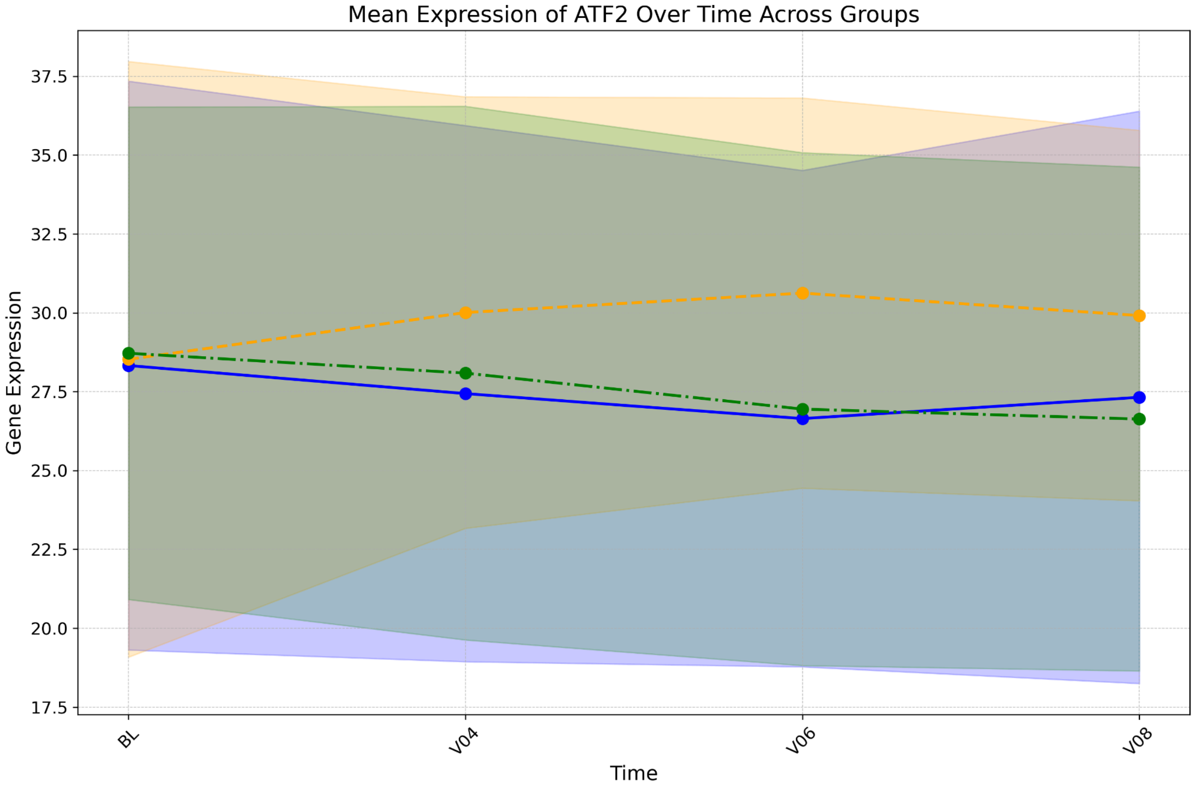
Mean Expression of ATF2 Over Time Across Groups
37.5
35.0
32.5
30.0
27.5
25.0
22.5
20.0
17.5
Gene Expression
BL
V04
V06
V08
Time

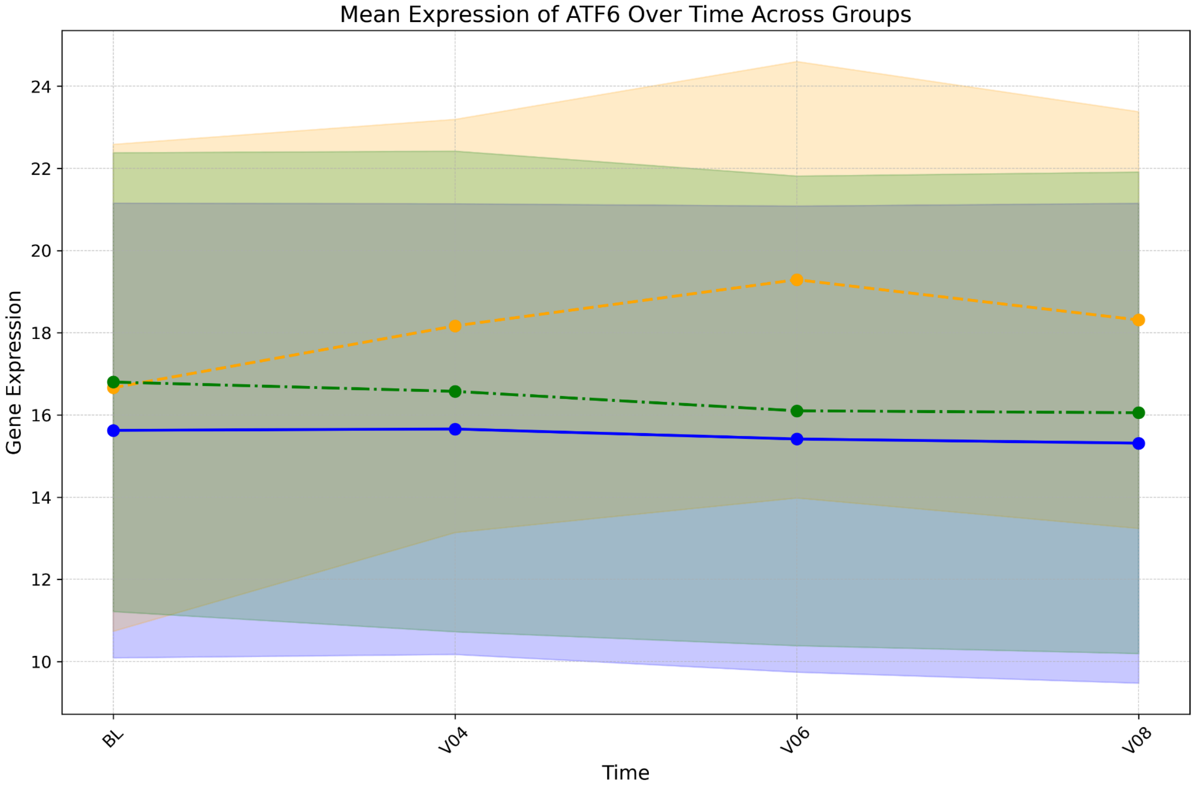
Mean Expression of ATF6 Over Time Across Groups
24
22
20
18
16
14
12
10
Gene Expression
BL
V04
V06
V08
Time

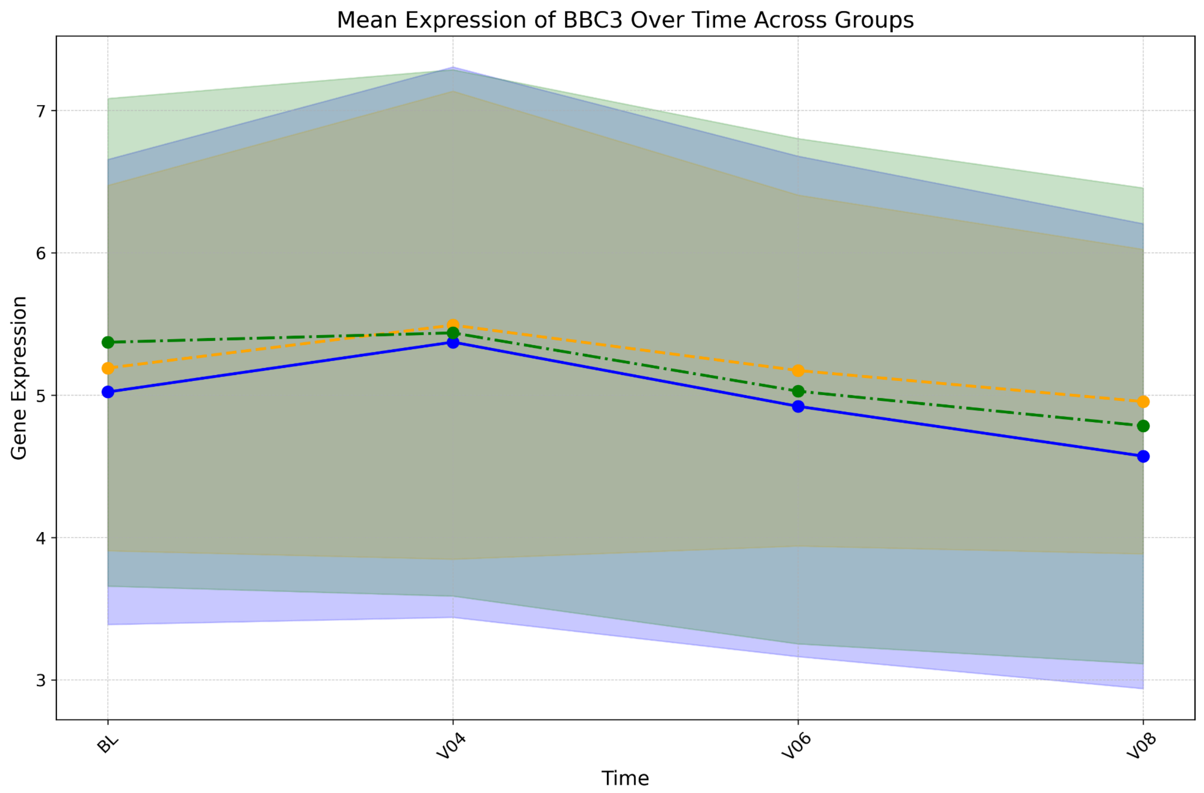
Mean Expression of BBC3 Over Time Across Groups
Gene Expression
Time
3
4
5
6
7
BL
V04
V06
V08

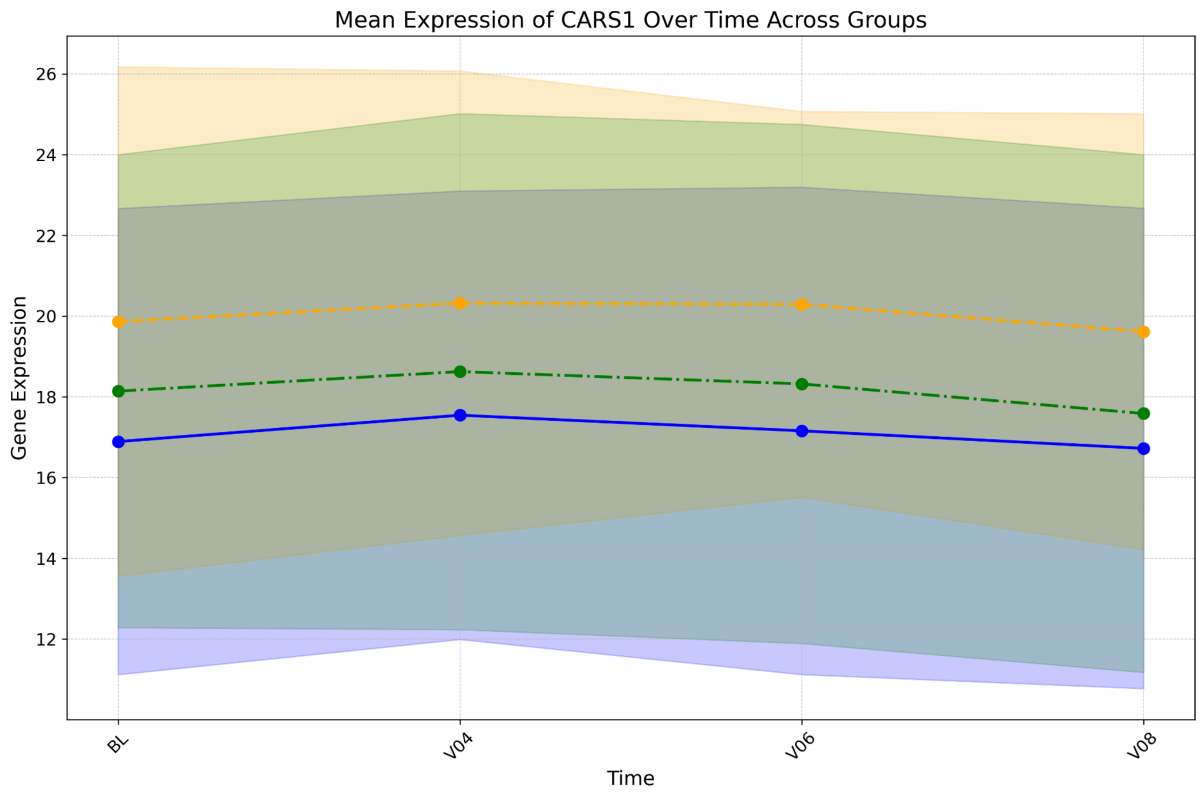
Mean Expression of CARS1 Over Time Across Groups
Gene Expression
Time
12
14
16
18
20
22
24
26
BL
V04
V06
V08

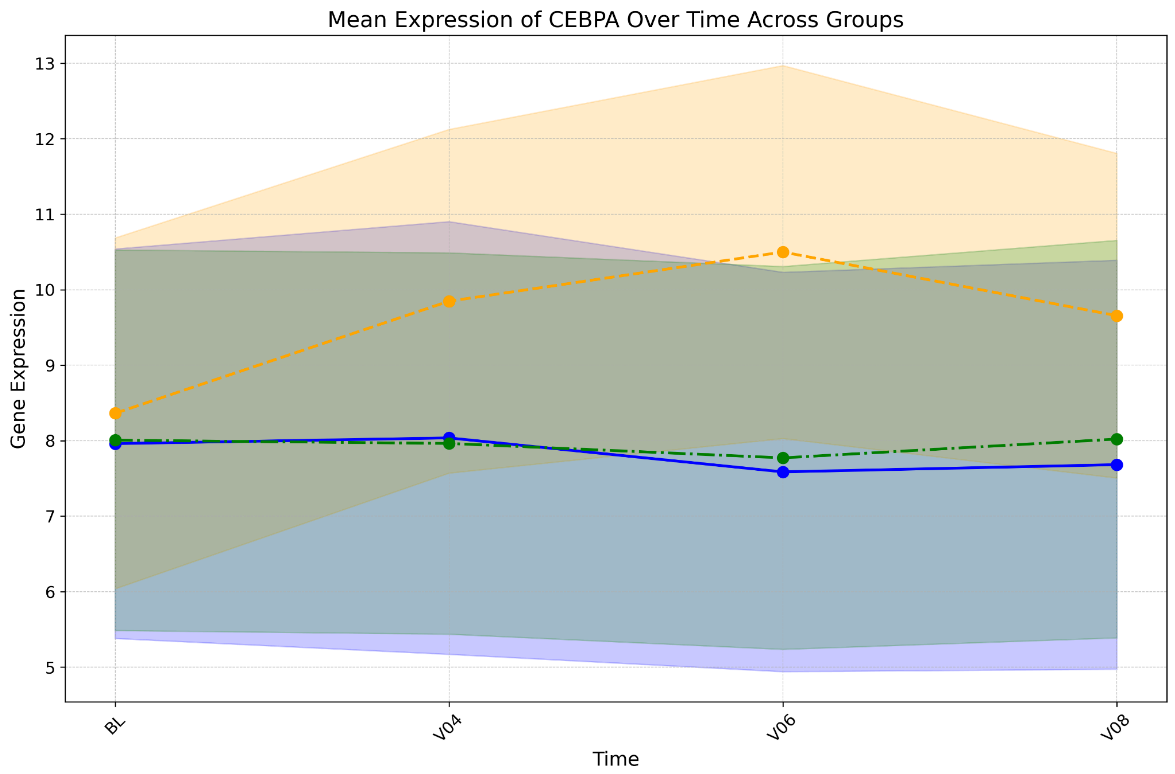
Mean Expression of CEBPA Over Time Across Groups
Gene Expression
Time
13
12
11
10
9
8
7
6
5
BL
V04
V06
V08

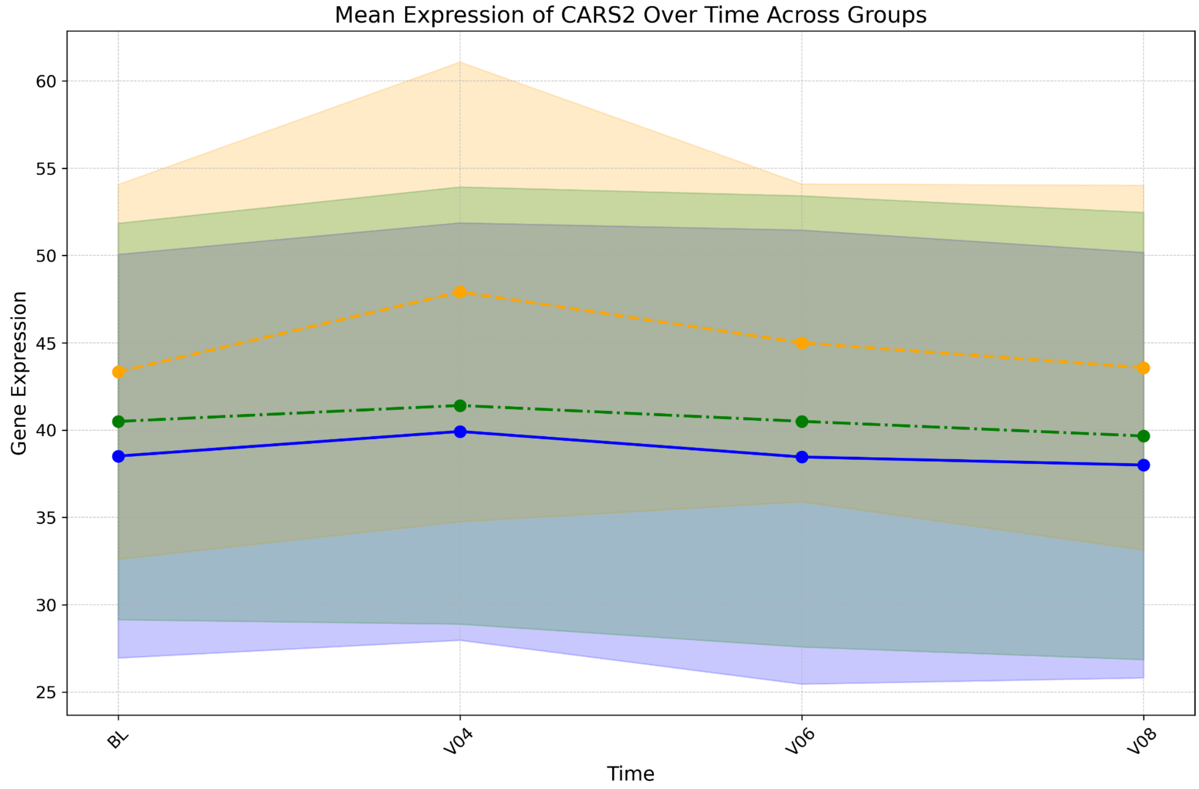
Mean Expression of CARS2 Over Time Across Groups
Gene Expression
Time
60
55
50
45
40
35
30
25
BL
V04
V06
V08

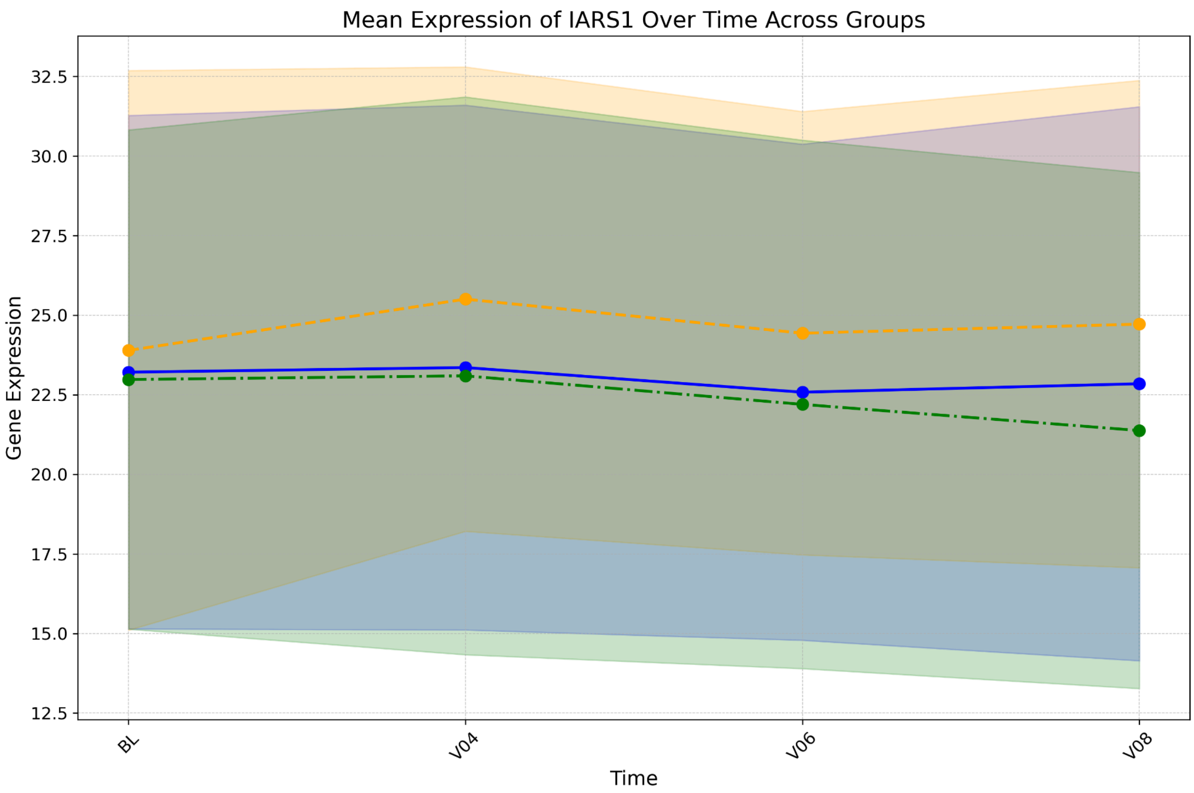

Mean Expression of IARS1 Over Time Across Groups
Gene Expression
32.5
30.0
27.5
25.0
22.5
20.0
17.5
15.0
12.5
BL
V04
V06
V08
Time

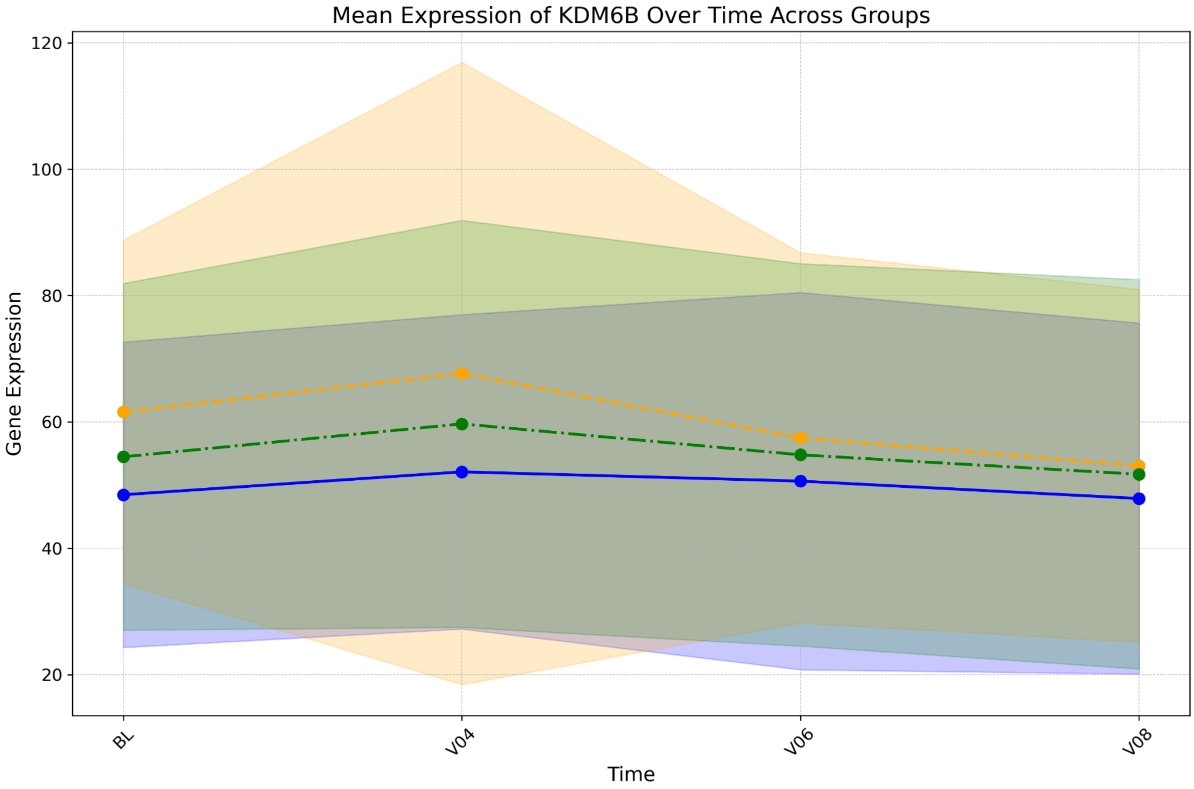

Mean Expression of KDM6B Over Time Across Groups
Gene Expression
120
100
80
60
40
20
BL
V04
V06
V08
Time

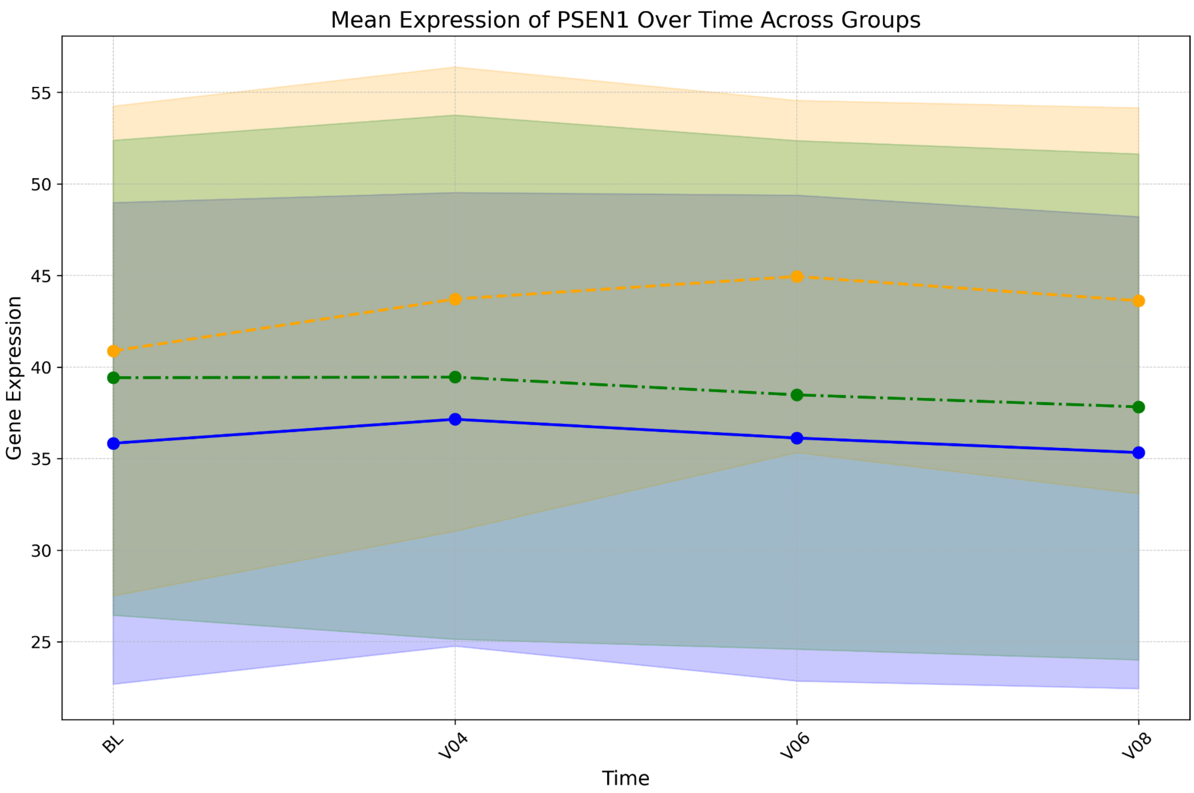
Mean Expression of PSEN1 Over Time Across Groups
Gene Expression
55
50
45
40
35
30
25
BL
V04
V06
V08
Time

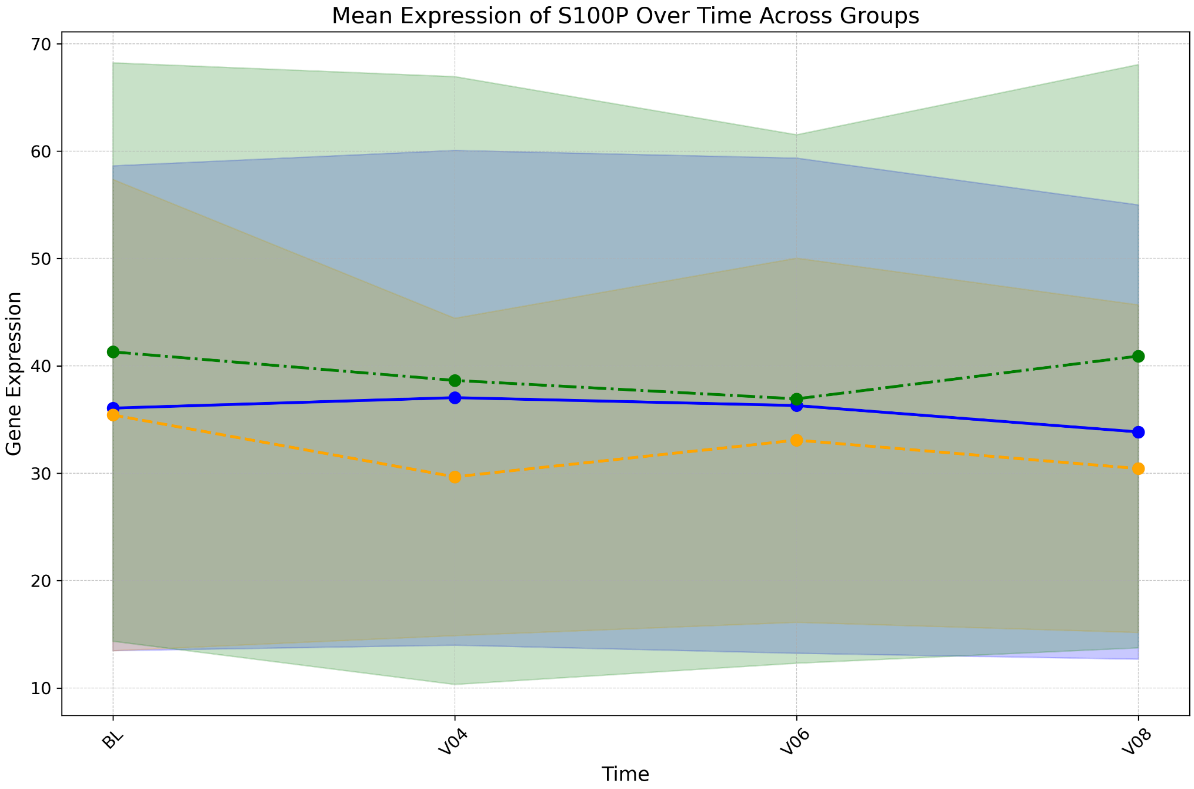
Mean Expression of S100P Over Time Across Groups
Gene Expression
70
60
50
40
30
20
10
BL
V04
V06
V08
Time

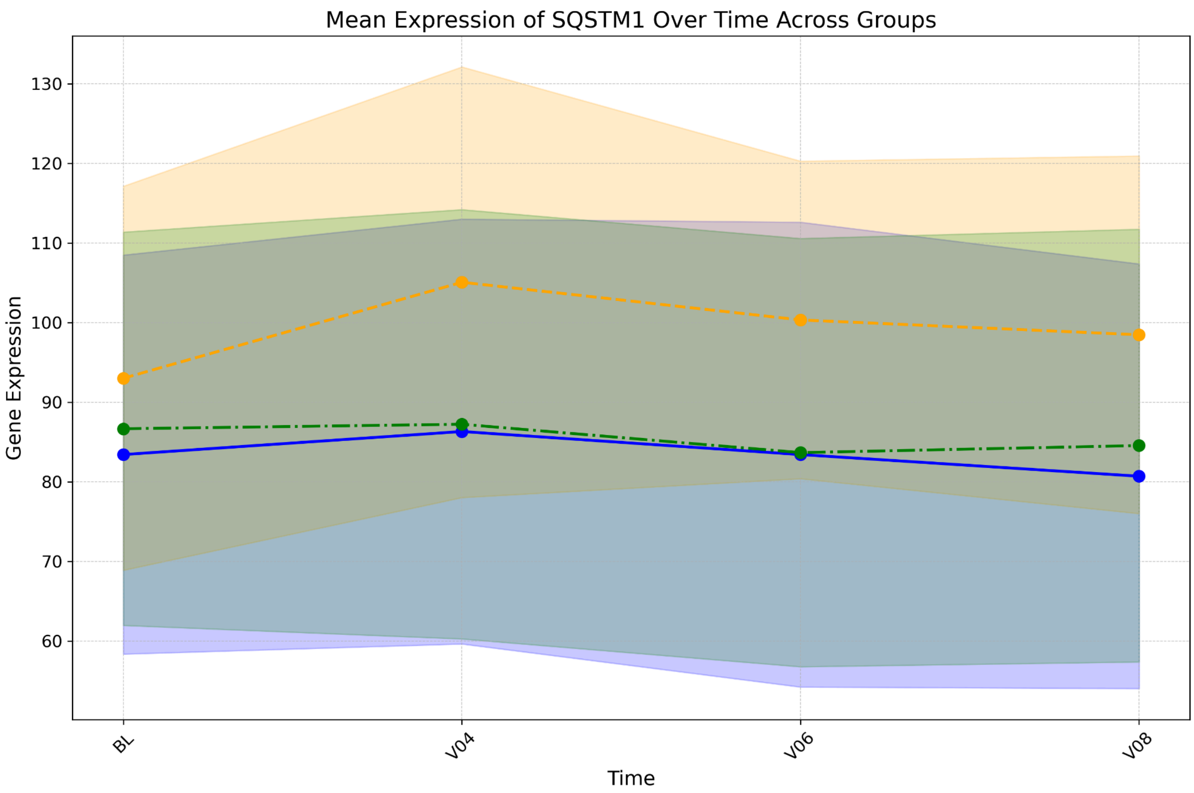

Mean Expression of SQSTM1 Over Time Across Groups
Gene Expression
130
120
110
100
90
80
70
60
BL
V04
V06
V08
Time
